# Small leucine-rich proteoglycans (SLRPs) in the endometrium of polycystic ovary syndrome women: a pilot study

**DOI:** 10.1186/s13048-017-0349-9

**Published:** 2017-08-08

**Authors:** Ricardo Santos Simões, José Maria Soares-Jr, Manuel J. Simões, Helena B. Nader, Maria Cândida P. Baracat, Gustavo Arantes R. Maciel, Paulo C. Serafini, Ricardo Azziz, Edmund C. Baracat

**Affiliations:** 1 Disciplina de Ginecologia, Departamento de Obstetrícia e Ginecologia, Hospital das Clínicas, Faculdade de Medicina da Universidade de São Paulo, São Paulo, Brazil; 2 Department of Molecular Biology, Federal University of São Paulo, São Paulo, Brazil; 3 Departments of Obstetrics and Gynecology and of Medicine, Medical College of Georgia, Augusta University, Augusta, GA USA; 4 Av. Dr. Enéas de Carvalho Aguiar, 255 - 10o.andar - Sala 10.167 - 05403-900, São Paulo, SP Brazil

**Keywords:** Polycystic ovary syndrome, Endometrium, Small leucine-rich proteoglycans, Proliferative phase

## Abstract

**Background:**

Small leucine-rich proteoglycans (SLRPs) play an important role in tissue homeostasis and cell proliferation since these proteoglycans sequester multiple growth factors. However, the content of SLRPs in the endometrium of polycystic ovary syndrome (PCOS) women is unknown. Our purpose was to test the hypothesis that excessive endometrial proliferation in PCOS may be partly related to abnormalities in SLRPs.

**Methods:**

In a cross section study a total of 20 endometrial samples were collected from 10 patients with PCOS and 10 ovulatory women during their proliferative (pre-ovulatory) phase. The study subjects were matched for age, body mass index and race. The age range was 20 to 35 years. All volunteers were evaluated in reproductive endocrinology clinic, Gynecology Division, Clinics Hospital, University of São Paulo Medical School Profile and concentration of small leucine-rich proteoglycans (decorin, lumican, fibromodulin and biglycan) were determined by immunohistochemical testing and Western blotting.

**Results:**

Decorin and lumican demonstrated higher immunoreactivity and relative expression in the endometrium of women with PCOS compared to that of women with regular menstrual cycles.

**Conclusion:**

Our data suggests that the endometrium of PCOS women demonstrate a greater content of SLRP than controls; decorin and lumican, in particular, were found in higher concentrations in the endometrium of PCOS women during the proliferative phase. These differences may, in part, explain the excess of endometrial proliferation frequently observed in PCOS. Further studies are warranted.

## Background

Polycystic ovary syndrome (PCOS) is an endocrine disorder which affects approximately 5 to 18% of women in childbearing age depending on the study population and the diagnostic criteria [1]. The syndrome is associated with ovulatory dysfunction, which can lead to menstrual cycle disruption including amenorrhea and oligomenorrhea increasing the incidence of infertility [2–4]. Additionally, in most cases PCOS show evidence of insulin resistance and hyperinsulinemia [1].

The incidence of endometrial hyperplasia in PCOS women is significantly higher than in controls and may, in part, be the result of hyperestrogenic anovulation and persistent hyperinsulinemia [5]. However, endometrial function in women with PCOS appears to differ significantly from that of normal endometrium, a difference that may predispose these patients to endometrial hyperplasia and carcinoma [6, 7].

Investigators have reported that in PCOS endometrial metabolism is altered, with local insulin resistance and increased growth factor content [8]. Growth factors mediating inflammation, including cytokines not only affect endometrial epithelial proliferation, but may alter factors in the extracellular matrix (ECM), including the production and action of small leucine-rich proteoglycans (SLRPs) [9, 10]. These proteoglycans play an important role in tissue homeostasis and cell proliferation, and protect the endometrium against agents that induce excessive endometrial proliferation [11–14]. Decorin, a specific SLRP, induces cell cycle arrest by activating cyclin-dependent protein kinase inhibitor, decreasing the activity and abundance of multiple cyclins, activating pro-apoptotic pathways and upregulating p21 synthesis in human endometrial stromal cells [14, 15].

The increased risk of endometrial hyperplasia and malignancy in PCOS is believed to be due not only to the reduction in the cyclic effect of progesterone, leading to chronic estrogen exposure, but also to the abnormal production of a large number of growth factors, cytokines, and other elements mediating inflammation [9, 10]. These factors may alter factors in the ECM, including SRLPs. However, the content of SLRPs in the endometrium of PCOS is unknown. Therefore, we designed a pilot study to test the hypothesis that the content of SLRPs, including decorin, lumican, fibromodulin, and biglycan, in the endometrium of PCOS subjects is detectable, and abnormal, which may predispose these patients to the development of endometrial hyperplasia and malignancy.

## Methods

### Study subjects

PCOS and control women were recruited from an outpatient reproductive endocrinology clinic at an academic tertiary medical care center (Gynecology Division, Clinics Hospital) from 2013 to 2015. We prospectively recruited all PCOS subjects first and then a match among controls was sought (i.e. ±4 kg/m^2^ in BMI, ±4 years in age, and similar race to the PCOS subject), either from a previously recruited pool of controls or, if no control with the needed parameters was so identified, then a new control was sought. This recruitment strategy yielded cohorts of PCOS and controls similar in number, mean BMI, mean age and race distribution.

#### PCOS patients

The diagnosis of PCOS was made in accordance with the Rotterdam 2003 criteria, and subjects were categorized by phenotype (phenotype A-D) [16, 17]. Clinical hyperandrogenism was established by the presence of hirsutism, determined by a modified Ferriman and Gallwey (mFG) score equal to or higher than 8.

#### Control

Controls were healthy women with confirmed fertility (having had at least one child previously), who had undergone tubal ligation and had a long-term history of menstrual cycles with regular intervals (25–35 days). These women were not using hormonal contraceptive methods.

All subjects (PCOS patients and control) were 20–35 years of age. Women who had causes of hyperandrogenism other than PCOS, anovulation which had been treated with hormones or other drugs in the 6 months prior to the study, systemic diseases, sexually transmitted diseases, uterine tumors, ovarian cysts or tumors, additional endocrine disorders, or using any hormones, including hormonally active herbal substances, statins, corticoids, or infertility drugs in the previous 6 months were excluded.

All participants of the study were subjected to a detailed history and physical examination, where height, weight, waist circumference (WC), hip circumference (HC), hirsutism by the mFG score, and a transvaginal ultrasound scan of the ovaries was obtained. Waist circumference was measured at the narrowest area between the last rib and the iliac crest, and HC at the most prominent point of the gluteal area.

This study was approved by the Research Ethics Committee of the Clinics Hospital, University of São Paulo School of Medicine (CEP 0164/09) and informed written consent was obtained from all subjects. The study was carried out from April 1, 2012 to August 31, 2015.

### Endometrial sampling

Endometrial samples (amount was 1.3 ± 0.45 g) were obtained using the Pipelle de Cornier catheter (Laboratoire CCD, Paris, France). Participants in the PCOS group were generally anovulatory and their endometrial biopsies were performed randomly after a negative pregnancy (β-hCG) result. Biopsies in the control group were scheduled during cycle days eight to twelve of the menstrual cycle. The rationale for this schedule was to avoid the menstruation period and the periovulatory phase [18].

### Histological processing of the endometrial samples

**Fig. 1:**
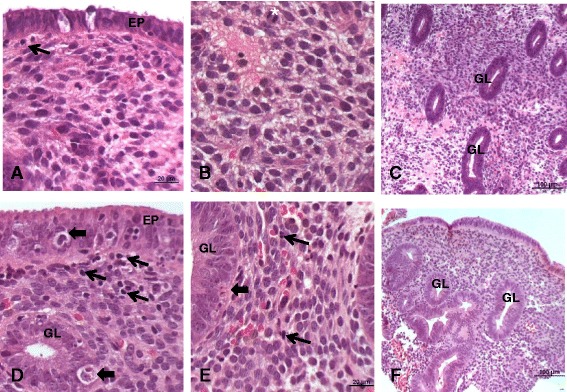
Photomicrographs of the endometrium in the proliferative phase (**a, b,** and **c**) of the CONTROL and PCOS women (**d, e,** and **f**). Note thicker surface epithelium (EP) and gland (GL) as well as a higher concentration of leukocytes within the lamina propria of the endometrium of PCOS patients in **d** and **e** compared to **a** and **b** (*thin arrows*). Also note typical apoptosis in **d** and **e** (*thick arrows*) (* mitotic figure). Staining with H.E. **d** = 200X, **b** and **e** = 400X, and **c** and **f** = 100X

Endometrial samples were fixed for 24 h in 10% formaldehyde, dehydrated in increasing concentrations of ethyl alcohol, cleared in xylene, and then imbedded in paraffin. Paraffin-embedded samples were then cut into ten 3-μm sections per subject. Eight of these sections were stained for SLRPs by immunohistochemistry (see below), and the two remaining sections were stained with hematoxylin and eosin (H&E), to confirm the menstrual cycle phase (proliferative) using the criteria suggested by Noyes et al. [19] (Fig. 1).

Sections of endometrium were incubated with the primary antibody for detection of decorin (Abcam 175,404), lumican (Abcam 168,348), fibromodulin (Bioss 12,362), and biglycan (Bioss 7552R), incubated with a second antibody, namely chicken antirabbit immunoglobulin G (DakoCytomation, Glostrup, Denmark), and then incubated in 0.05% DAB (3,3′-diaminobenzidine) (Sigma Chemical Co). Negative controls were prepared by incubating the sections with rabbit nonspecific immunoglobulin fraction (DAKOCytomation) instead of the primary antibody and by staining the sections with hematoxylin for nucleous identification.

The images obtained were examined with optical microscope and digitally analyzed with the Carl Zeiss® AxioVision microscopy software (Rel. 4.6. Oberkochen, Baden-Württemberg).

### Tissue homogenate preparation and Western blot analysis

Endometrial samples were homogenized, prepared, and sonicated in a buffer of 50 mM Tris-HCl, 150 mM NaCl, and 1% Triton-X pH 7.4 containing a complete protease inhibitor cocktail and PhosSTOP tablets (Roche Applied Science). Protein levels were determined by the Bradford assay and equalized prior to boiling in Laemmli buffer (Bio-Rad Laboratories, Hercules, CA, USA). To detect Gal-1 and MAPKs, protein extracts (40 μg per lane) were loaded onto a 15% SDS-PAGE together with appropriate molecular weight markers (Bio-Rad Laboratories) and transferred to ECL Hybond nitrocellulose membranes. Reversible protein staining of the membranes with 0.1% Ponceau-S in 5% acetic acid (Santa Cruz Biotechnology, Santa Cruz, CA, USA) was performed to verify protein transfer.

Membranes were incubated for 15 min in 5% BSA in Tris-buffered saline (TBS) before the addition of the rabbit polyclonal antibodies anti-decorin (Abcam175404), anti-lumican (Abcam168348), anti-fibromodulin (Bioss-12362R), anti-biglycan (Bioss-7552R), and anti-β-actin (Bs-12362R), all of which were diluted 1:500 in TBS with 0.1% Tween 20 (antibodies were purchased from Santa Cruz Biotechnology). Incubation was followed by 15 min of washing with TBS and 60 min of incubation at room temperature with peroxidase-conjugated goat anti-rabbit IgG (1:1000; Thermo Fisher Scientific, Inc., Rockford, MI, USA). Membranes were again washed for 15 min with TBS. Immunoreactive proteins were detected using an enhanced Supersignal West Pico chemiluminescent substrate kit (Thermo Fisher Scientific).

After exposure of the blots in dark room the resulting x-ray film was developed, imaged and quantified using ImageJ software1.440 (http://imagej.nih.gov/ij/ National Institutes of Health, Bethesda, MD, USA) to determine the relative expression (arbitrary units, AU) of SRPL/β-actin.

### Hormonal assays

The serum levels of thyroid-stimulating hormone (TSH), free thyroxine, follicle-stimulating hormone (FSH), lutein hormone (LH), total testosterone (TT), free testosterone (Tfree), androstenedione (A), cortisol, prolactin (PRL), dehydroepiandrosterone sulfate (DHEAS), 17α-hydroxyprogesterone (17-OHP), fasting insulin, fasting glucose, 2-h oral glucose tolerance test, β-human chorionic gonadotropin (β-hCG), and sex hormone-binding globulin (SHBG) were measured in both groups. Total testosterone levels were determined in triplicate using an electrochemiluminescence immunoassay (Testosterone II Cobas™ kit, Roche Diagnostics GmbH, Mannheim, Germany). The minimum detectable level of TT with this assay is 0.025 ng/ml and the main cross-reaction is with androstenedione (< 2.5%). The intra assay coefficient of variation (CV) was 12.5% and the inter assay CV was 13.8%.

### Statistical analysis

All measurements were assessed for normality using the Kolmogorov-Smirnov test. The unpaired Student t-test was used to compare the PCOS and CONTROL groups. Statistical analysis was performed using the GraphPad Prism software version 3.0 for Windows (GraphPad Software, San Diego, CA, USA). The data are presented as mean ± standard deviation (SD), and the significance level was set at *p* < 0.05.

## Results

A total of 20 women, aged 20 to 35 years, agreed to participate in this pilot study: a) 10 women with PCOS (PCOS), all phenotype A (hyperandrogenism either clinical and/or biochemical + ovulatory dysfunction + polycystic ovarian morphology), and b) 10 matched controls (CONTROL). The characteristics of these groups are depicted in Table 1.

**Table 1:** Characteristics of participants

	CONTROL (*n* = 10)	PCOS (*n* = 10)	*p*-value
Age, years	28.6 ± 2.0	29.6 ± 1.2	NS
Caucasian, n	5	6	NS
BMI, Kg/m^2^	27.49 ± 2.45	28.01 ± 3.19	NS
TT (ng/dL)	35.9 ± 11.7	83.09 ± 24.8	< 0.05*
DHEAS (mg/mL)	421.5 ± 52.1	520.4 ± 82.1	NS
TSH (μUI/mL)	2.8 ± 1.7	2.9 ± 1.6	NS
Insulin (μUI/mL)	8.8 ± 2.9	15.5 ± 3.5	< 0.05*
Glucose (mg/dL)	81.8 ± 8.6	89.2 ± 11.9	NS
17-OHP (mg/mL)	17.3 ± 8.4	18.3 ± 7.4	NS
FSH (mUI/ml)	5.0 ± 1.2	5.1 ± 1.1	NS
LH (mUI/ml)	5.9 ± 2.9	9.5 ± 3.8	< 0.05*
PRL (ng/dl)	10.8 (4.3–34.5)	11.4 (4.2–33.5)	NS
A (ng/mL)	2.2 ± 0.8	4.2 ± 1.3	< 0.05*
Tfree (pg/dl)	1.7 ± 0.40	2.1 ± 1.0	NS

Data are expressed as median and standard deviation; *BMI* Body mass index, *TT* total testosterone, *TSH* thyroid stimulating hormone, *Tfree* free testosterone, *A* androstenedione, *LH* lutein hormone, *FSH* follicle-stimulating hormone, *PRL* prolactin, *17-OHP* 17α-hydroxyprogesterone, *DHEAS* dehydroepiandrosterone sulfate, *NS* nonsignificant difference (*p* > 0.05). *Significance of *P*-value <0.05

### Morphological analysis of endometrial samples

Morphological analysis confirmed that in CONTROL endometrial tissue obtained was in the proliferative phase on days 8–12 of the menstrual cycle. Samples demonstrated a simple columnar epithelium lining and well-developed glands with cells in mitosis and endometrial stroma with several fibroblasts with an obvious elliptical nucleus, indicating intense cellular activity and synthesis of extracellular matrix components **(**Fig. 1 **)**, which are normal findings in the proliferative phase of the menstrual cycle in ovulatory patients.

The endometrial tissue of PCOS subjects had the epithelial lining and the glands were thicker, and the endometrial stroma contained many leukocytes, consistent with excessive estrogenic action compared to CONTROL subjects [20]. In some PCOS subjects epithelial cells with signs of apoptosis and well-developed endometrial stroma were observed (Fig. 1).

### Immunohistochemical detection and comparison of SLRPs in the endometrium

Immunohistochemical analysis demonstrated SLRPs throughout the ECM of endometrial samples of both CONTROL and PCOS subjects. In both groups, SLRPs were also present throughout the endometrial stroma and could be observed clearly in the basement membrane of blood vessels, the epithelial lining, and the glands.

**Fig. 2:**
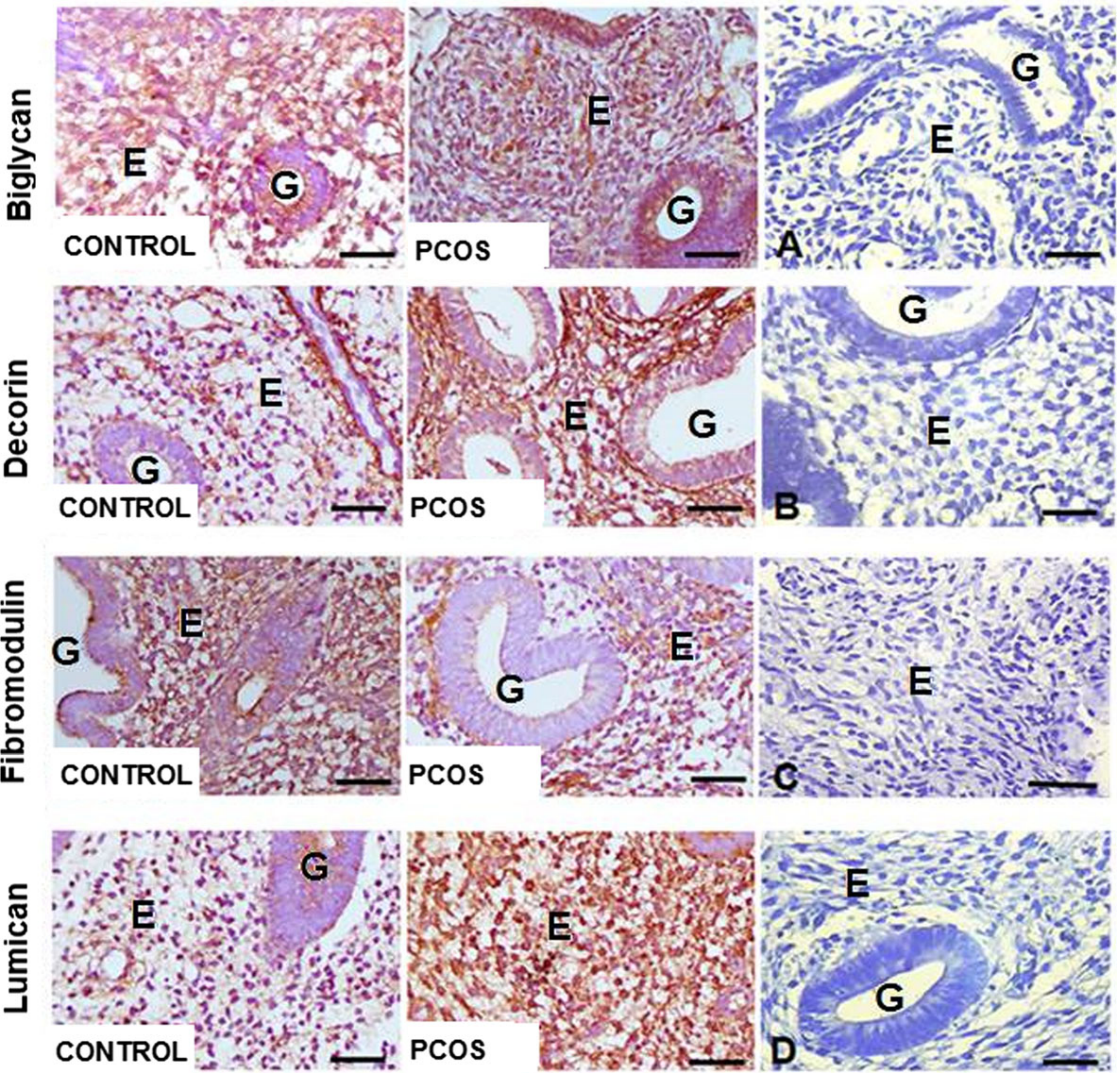
Photomicrographs of the endometrium in the proliferative phase of CONTROL and PCOS women. Endometrial fragments subjected to immunohistochemical methods for identification of decorin, biglycan, lumican, and fibromodulin. Bar = 20 μm

In CONTROL the intensity of immunoreactivity of biglycan and lumican was increased compared to that of decorin and fibromodulin. Alternatively, in PCOS biglycan, decorin, and lumican immunoreactivity were heightened compared with that of fibromodulin. Overall, the immunoreactivity pattern of SRPLs was higher in PCOS compared to CONTROL subjects (Fig. 2).

### Quantification of SLRP expression by Western blotting

**Fig. 3:**
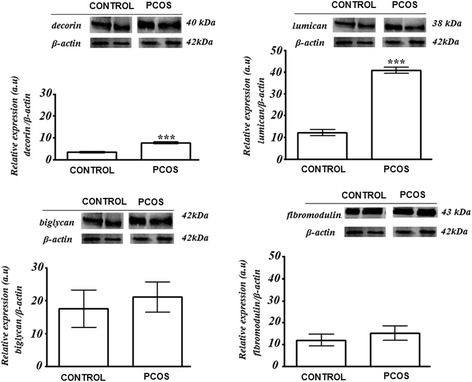
Western blotting results for decorin, lumican, biglycan, and fibromodulin expression in the endometrium of CONTROL and PCOS women

The expression of each SRPL studied (i.e. decorin, lumican, fibromodulin, and biglycan) relative to that of β-actin is summarized in Table 2 and Fig. 3. Overall, decorin and lumican had higher relative expressions in the PCOS than in CONTROL subjects (*p* < 0.05).

**Table 2:** SLRP content in endometrial stromal cells in PCOS and CONTROL subjects^a^

	Anti-decorin	Anti-biglycan	Anti-lumican	Anti-fibromodulin
CONTROL	3.4 ± 0.3**	17.5 ± 5.7	12.1 ± 0.5**	11.9 ± 2.7
PCOS	7.6 ± 0.5	21.1 ± 4.6	40.7 ± 0.4	15.1 ± 3.2

^a^Western blotting yield relative to beta-actin values of the SLRPs studied (mean ± SD) in endometrial stromal cells. Data are expressed as median and standard deviation. ** Significance of *P*-value <0.05

## Discussion

Endometrial growth and differentiation is under the influence of sex hormones such as estrogen, progesterone, and androgens. In women with PCOS there is a deficiency in cyclic progesterone production due to chronic anovulation and a relative increase in other substances, such as insulin, cytokines, and growth factors, which results in an imbalance in endometrial hemostasis in these patients, possibly leading to abnormal uterine bleeding, infertility, and an increased risk of developing endometrial hyperplasia [3, 21–25]. The specific receptors for insulin and Insulin Growth Factors (IGFs) as well as high-affinity IGF-binding proteins (IGFBPs) have been detected in endometrial tissues [26]. IGFs have proliferative effects and the secretion of these IGFBPs, which can modulate the bioavailability of IGFs by competing with the IGF receptor, is stimulated by progesterone and inhibited by insulin. Consequently the action of IGFs may be more intense in endometrial cells [6]. Moreover, both insulin and IGF-I inhibit the hepatic synthesis of sex-hormone binding globulin (SHBG) [27], and insulin stimulates aromatase activity in both endometrial glands and stroma, thus enhancing endogenous endometrial estrogen production [27, 28]. The final result is an excess of estrogen and IGFs action on the endometrium of PCOS.

Furthermore, data on stromal (non-epithelial) structures in the endometrium of women with PCOS are lacking [3, 10, 24, 25]. Therefore, our pilot study has contributed to the scientific literature with the following two findings: a) demonstrating the presence of SLRPs in the endometrium of women with PCOS; and b) demonstrating that the SLRP content in these women appears to be abnormal, specifically appearing to be enhanced relative to normals.

Previous studies have reported that SRLPs in the endometrium are important for embryonic implantation and placentation [24, 29, 30]. However, such studies have not included women with PCOS. In fact, the expression and distribution of endometrial decorin, biglycan, lumican, and fibromodulin are hormone dose dependent [30]. The hyperandrogenism may explain the upregulated production of decorin and lumican. Under the influence of the ovarian hormones estrogen and progesterone, the endometrial SLRPs undergo profound changes during the menstrual cycle in normal women. However, we have not assayed the level of estrogen and progesterone at time of endometrial sampling. It is one limitation of our study.

Increased decorin and lumican in endometrial ECM may have a local antiproliferative activity due to bind cytokines and growth factors [31, 32]. Furthermore, some investigators suggest that decorin and lumican inhibit tumor growth, particularly in prostate and lung cancer [33, 34]. Thus, these SLRPs may partially protect the endometrium of women with PCOS from malignant transformation.

The mechanism of action of decorin and lumican includes blocking tyrosine kinase receptors (TKR) and reducing the influence of EGFR, IGF-IR, HGFR, and VEGFR-2 on the endometrium. Moreover, decorin restrains angiogenesis by binding to thrombospondin-1, TGFβ, VEGFR-2, and possibly IGF-IR, and then halting tumor growth by antagonizing oncogenic TKRs and angiogenesis [35, 36]. These effects may negatively act on the implantation process by inhibiting trophoblast migration and invasion [36].

The collagen fiber is the most abundant component in the extracellular matrix. It is present in the ECM as a fibrillar protein providing structural support to cells and participating in cell-cell and cell-matrix interactions; thus, it has a fundamental role in tissue architecture [37–39]. Collagen fibers interact with extracellular matrix components, such as heparin sulfate. A research carried out by Giordano et al. [40] demonstrate alterations in the endometrial glycosaminoglycan levels – chiefly those of heparan sulfate – in women with PCOS.

## Conclusion

Notwithstanding the sex steroid disorder, the exact pathophysiological mechanisms of endometrial PCOS believed to account for excessive endometrial proliferation are not known [41, 42]. Our data suggests that the endometrium of patients with PCOS exhibits higher expressions of decorin and lumican than that of healthy control women in the proliferative phase of the menstrual cycle. It is then possible that this proteoglycan excess may interfere with normal endometrial hemostasis in PCOS. Further studies are warranted.

## Abbreviations

17-OHP: 17α-hydroxyprogesterone
A: Androstenedione
CV: Coefficient of variation
DHEAS: Dehydroepiandrosterone sulfate
ECM: Extracellular matrix
EGFR: Epidermal growth factor receptor
FSH: Follicle-stimulating hormone
H&E: Hematoxylin and eosin
HC: Hip circumference
IGFIR: Insulin-like growth factor receptor I
LH: Lutein hormone
MET: Hepatocyte growth factor receptor
mFG: Modified Ferriman and Gallwey
PCOS: Polycystic ovarian syndrome
PRL: Prolactin
SHBG: Sex hormone-binding globulin
SLRPs: Small leucine-rich proteoglycans
TBS: Tris-buffered saline
Tfree: Free testosterone
TGF: Transforming growth factor
TKR: Tyrosine kinase receptors
TSH: Thyroid-stimulating hormone
TT: Total testosterone
WC: Waist circumference
β-hCG: β-human chorionic gonadotropin

## References

[1] Azziz R (2016). Introduction: Determinants of polycystic ovary syndrome. Fertil Steril.

[2] Hull MG (1987). Epidemiology of infertility and polycystic ovarian disease: endocrinological and demographic studies. Gynecol Endocrinol.

[3] Lopes IM, Maganhin CC, Oliveira-Filho RM, Simões RS, Simões MJ, Iwata MC (2014). Histomorphometric Analysis and Markers of Endometrial Receptivity Embryonic Implantation in Women With Polycystic Ovary Syndrome During the Treatment With Progesterone. Reprod Sci.

[4] Balen AH, Conway GS, Kaltsas G, Techatrasak K, Manning PJ, West C, Jacobs HS (1995). Polycystic ovary syndrome: the spectrum of the disorder in 1741 patients. Hum Reprod.

[5] Barry JA, Azizia MM, Hardiman PJ (2014). Risk of endometrial, ovarian and breast cancer in women with polycystic ovary syndrome: a systematic review and meta-analysis. Hum Reprod Update.

[6] Gadducci A, Gargini A, Palla E, Fanucchi A, Genazzani AR (2005). Polycystic ovary syndrome and gynecological cancers: is there a link?. Gynecol Endocrinol.

[7] Pillay OC, Te Fong LF, Crow JC, Benjamin E, Mould T, Atiomo W, Menon PA, Leonard AJ, Hardiman P (2006). The association between polycystic ovaries and endometrial cancer. Hum Reprod.

[8] Roemer KL, Young SL, Savaris RF (2014). Characterization of GAB1 expression over the menstrual cycle in women with and without polycystic ovarian syndrome provides a new insight into its pathophysiology. J Clin Endocrinol Metab.

[9] Lopes IM, Baracat MC, Simões Mde J, Simões RS, Baracat EC, Soares JM (2011). Endometrium in women with polycystic ovary syndrome during the window of implantation. Rev Assoc Med Bras.

[10] Baracat MC, Serafini PC, Simões Rdos S, Maciel GA, Soares JM, Baracat EC (2015). Systematic review of cell adhesion molecules and estrogen receptor expression in the endometrium of patients with polycystic ovary syndrome. Int J Gynaecol Obstet.

[11] Mauviel A, Santra M, Chen YQ, Uitto J, Iozzo RV (1995). Transcriptional regulation of decorin gene expression. Induction by quiescence and repression by tumor necrosis factor-alpha. J Biol Chem.

[12] Seidler DG, Dreier R (2008). Decorin and its galactosaminoglycan chain: extracellular regulator of cellular function?. IUBMB Life.

[13] Iozzo RV (1997). The family of the small leucine-rich proteoglycans: key regulators of matrix assembly and cellular growth. Crit Rev Biochem Mol Biol.

[14] Iozzo RV, Sanderson RD (2011). Proteoglycans in cancer biology, tumour microenvironment and angiogenesis. J Cell Mol Med.

[15] Ono YJ, Terai Y, Tanabe A, Hayashi A, Hayashi M, Yamashita Y, Kyo S, Ohmichi M (2014). Decorin induced by progesterone plays a crucial role in suppressing endometriosis. J Endocrinol.

[16] Rotterdam ESHRE/ASRM-Sponsored PCOS Consensus Workshop Group (2004). Revised 2003 consensus on diagnostic criteria and long-term health risks related to polycystic ovary syndrome (PCOS). Hum Reprod.

[17] Lizneva D, Suturina L, Walker W, Brakta S, Gavrilova-Jordan L, Azziz R (2016). Criteria, prevalence, and phenotypes of polycystic ovary syndrome. Fertil Steril.

[18] Fraser IS, Critchley HO, Broder M, Munro MG (2011). The FIGO recommendations on terminologies and definitions for normal and abnormal uterine bleeding. Semin Reprod Med.

[19] Noyes RW, Hertig AT, Rock J (1975). Dating the endometrial biopsy. Am J Obstet Gynecol.

[20] Andrade PM, Baracat EC, Simões MJ, Rodrigues de Lima G (2000). Histomorphometric aspects of adult castrated rat endometrium after the use of estrogen, progesterone and tamoxifen. Clin Exp Obstet Gynecol.

[21] Cheung AP (2001). Ultrasound and menstrual history in predicting endometrial hyperplasia in polycystic ovary syndrome. Obstet Gynecol.

[22] Apparao KBC, Lovely LP, Gui Y, Lininger RA, Lessey BA (2002). Elevated endometrial androgen receptor expression in women with polycystic ovarian syndrome. Biol Reprod.

[23] San Martin S, Soto-Suazo M, Ferreira de Oliveira S, Aplin JD, Abrahamsohn P, TMT Z (2003). Small leucine-rich proteoglycans (SLRPs) in uterine tissues during pregnancy in mice. Reproduction.

[24] Giudice LC (2006). Endometrium in PCOS: Implantation and predisposition to endocrine CA. Best Pract Res Clin Endocrinol Metab.

[25] Iatrakis G, Tsionis C, Adonakis G, Stoikidou M, Anthouli-Anagnostopoulou F, Parava M (2006). Polycystic ovarian syndrome, insulin resistance and thickness of the endometrium. Eur J Obstet Gynecol Reprod Biol.

[26] Rutanen EM (1998). Insulin-like growth factors in endometrial function. Gynecol Endocrinol.

[27] Kaaks R (2001). Plasma insulin, IGF-I and breast cancer. Gynecol Obstet Fertil.

[28] Randolph JF, Kipersztok S, Ayers JW, Ansbacher R, Peegel H, Menon KM (1987). The effect of insulin on aromatase activity in isolated human endometrial glands and stroma. Am J Obstet Gynecol.

[29] Kitaya K, Yasuo T (2009). Dermatan sulfate proteoglycan biglycan as a potential selectin L/CD44 ligand involved in selective recruitment of peripheral blood CD16(−) natural killer cells into human endometrium. J Leukoc Biol.

[30] Salgado RM, Favaro RR, Zorn TM (2011). Modulation of small leucine-rich proteoglycans (SLRPs) expression in the mouse uterus by estradiol and progesterone. Reprod Biol Endocrinol.

[31] Yamaguchi Y, Ruoslahti E (1988). Expression of human proteoglycan in Chinese hamster ovary cells inhibits cell proliferation. Nature.

[32] Vij N, Roberts L, Joyce S, Chakravarti S (2004). Lumican suppresses cell proliferation and aids Fas - Fas ligand mediated apoptosis: implications in the cornea. Exp Eye Res.

[33] Niu C, Liang C, Guo J, Cheng L, Zhang H, Qin X, Zhang Q, Ding L, Yuan B, Xu X (2012). Downregulation and growth inhibitory role of FHL1 in lung cancer. Int J Cancer.

[34] Coulson-Thomas VJ, Coulson-Thomas YM, Gesteira TF, Andrade de Paula CA, Carneiro CR, Ortiz V (2013). Lumican expression, localization and antitumor activity in prostate cancer. Exp Cell Res.

[35] Hildebrand A, Romarís M, Rasmussen LM, Heinegård D, Twardzik DR, Border WA, Ruoslahti E (1994). Interaction of the small interstitial proteoglycans biglycan, decorin and fibromodulin with transforming growth factor beta. Biochem J.

[36] Lala PK, Nandi P (2016). Mechanisms of trophoblast migration, endometrial angiogenesis in preeclampsia: The role of decorin. Cell Adhes Migr.

[37] Vogel KG, Paulsson M, Heinegard D (1984). Specific inhibition of type I and type II collagen fibrilogenesis by the small proteoglycan of tendon. Biochem J.

[38] Vogel KG, Trotter JA (1987). The effect of proteoglycan on the morphology of collagen fibrils formed in vitro. Coll Relat Res.

[39] Rada JA, Cornuet PK, Hassell JR (1993). Regulation of corneal collagen fibrillogenesis in vitro by corneal proteoglycan (lumican and decorin) core proteins. Exp Eye Res.

[40] Giordano MV, Giordano LA, Gomes RC, Simões RS, Nader HB, Giordano MG (2015). The evaluation of endometrial sulfate glycosaminoglycans in women with polycystic ovary syndrome. Gynecol Endocrinol.

[41] Azziz R, Carmina E, Dewailly D, Diamanti-Kandarakis E, Escobar-Morreale HF, Futterweit W (2006). Positions statement: criteria for defining polycystic ovary syndrome as a predominantly hyperandrogenic syndrome: an Androgen Excess Society guideline. J Clin Endocrinol Metab.

[42] Christ JP, Willis AD, Brooks ED, Vanden Brink H, Jarrett BY, Pierson RA, Chizen DR, Lujan ME (2014). Follicle number, not assessments of the ovarian stroma, represents the best ultrasonographic marker of polycystic ovary syndrome. Fertil Steril.

